# Orthopedic Management of Patients with Pompe Disease: A Retrospective Case Series of 8 Patients

**DOI:** 10.1155/2014/963861

**Published:** 2014-01-02

**Authors:** Gerrit Haaker, Jürgen Forst, Raimund Forst, Albert Fujak

**Affiliations:** Department of Orthopedic Surgery, Friedrich-Alexander-University Erlangen-Nuremberg, Rathsberger Straße 57, 91054 Erlangen, Germany

## Abstract

*Introduction.* Pompe disease (PD), a lysosomal storage disease as well as a neuromuscular disorder, is a rare disease marked by progressive muscle weakness. Enzyme replacement therapy (ERT) in recent years allowed longer survival but brought new problems to the treatment of PD with increasing affection of the musculoskeletal system, particularly with a significantly higher prevalence of scoliosis. The present paper deals with the orthopedic problems in patients with PD and is the first to describe surgical treatment of scoliosis in PD patients. *Patients and Methods.* The orthopedic problems and treatment of eight patients with PD from orthopedic consultation for neuromuscular disorders are retrospectively presented. We analyzed the problems of scoliosis, hip dysplasia, feet deformities, and contractures and presented the orthopedic treatment options. *Results.* Six of our eight PD patients had scoliosis and two young patients were treated by operative spine stabilization with benefits for posture and sitting ability. Hip joint surgery, operative contracture release, and feet deformity correction were performed with benefits for independent activity. *Conclusion.* Orthopedic management gains importance due to extended survival and musculoskeletal involvement under ERT. Surgical treatment is indicated in distinct cases. Further investigation is required to evidence the effect of surgical spine stabilization in PD.

## 1. Introduction

Pompe disease (PD), glycogen-storage disease type II or acid maltase deficiency, is a very rare autosomal recessive disorder due to dysfunction of the enzyme acid alpha glucosidase. PD is at once a glycogen and lysosomal storage disorder as well as a neuromuscular disorder, marked by progressive muscle weakness [1, 2]. The estimated overall incidence of PD average is 1 : 40000 [3, 4].

PD includes a wide range of symptoms and has a variable classification. The disease is commonly divided into infantile-onset (IOPD) and late-onset (LOPD) PD [1, 5, 6]. The more severe infantile-onset form occurs within the first year of life and is characterized by hypotonia (floppy infant), feeding difficulties, massive respiratory dysfunction, and death occurring by two years of age due to cardiorespiratory failure [1]. Nowadays, a distinction is usually drawn between the classic infantile-onset form with distinct cardiomyopathy and the so-called atypical or muscular infantile-onset form without or with less severe cardiac involvement [2].

The late-onset form has symptoms developing anytime after the first year of life and it is subdivided into childhood- (2–13 years), juvenile- (13–20 years), and adult- (>20 years) onset forms [5, 6]. LOPD is characterized by progressive muscle weakness occurring throughout the body as well as restrictive respiratory dysfunction sporadically in combination with cardiac involvement. The severity of the development of LOPD can vary widely [1, 2, 6].

A milestone in the treatment of PD was reached in 2006, when enzyme replacement therapy (ERT) was accredited and now offers a first primary therapy of the disease mechanism. The success of ERT was recently shown in different publications [2, 7–10] with effects on skeletal muscle weakness progression, cardiac involvement, and respiratory function. The ventilator-free period was extended and patients gained functional independence, retained their sitting stability or independent walking ability, and reached new motor milestones. The mean survival was shown to be significantly higher with ERT [2, 8, 10, 11].

The success of the ERT [7] brought new problems to the management of PD. New symptoms arose, causing a different phenotype which cannot only be explained by a possible shift from infantile to late-onset phenotype due to the reduction of symptoms with the ERT [12]. Recent publications have shown that a main part of the new phenotype symptoms is caused by progressive muscle weakness in spite of successful ERT [13, 14]. During long-term observation of PD patients treated by ERT, the problems most often described are facial weakness, ptosis, hip extensor and dorsiflexor weakness, gait and posture difficulties, and, especially, scoliosis [13, 14].

Against this background, we describe eight patients with PD with a wide range of disease onset and severity from the orthopedic point of view. We specify the conservative and operative orthopedic treatment possibilities, especially the management of scoliosis. Although scoliosis is a common complication of PD [15] and its prevalence is increasing with the success of enzyme replacement therapy [1, 6, 15], the orthopedic treatment of scoliosis in connection with PD is only rarely described [2, 15].

## 2. Patients and Methods

Eight patients, aged, 3 to 59 (mean 23,1) years diagnosed with PD by muscle biopsy were treated during special orthopedic consultations for patients with neuromuscular disorders. There were seven males and one female patient. The patients were interviewed and examined formally during our special muscle-disease consultations. We reviewed the patient data retrospectively from our surgeries and from patient records concerning disease onset and diagnosis, ERT therapy, sitting, standing, and walking abilities, musculoskeletal symptoms, respiratory involvement, and the surgical and conservative orthopedic treatment. Longtime follow-up was very restricted as the patients were partly present only for a particular orthopedic intervention in our neuromuscular disease center with follow-up at their local hospital. Limited and fragmental follow-up data was available for 6 of 8 patients and ranged from 14 months to 10 years.

This study is in accordance with the ethical standards of the relevant local Ethics Commission (Research Ethics Committee, Faculty of Medicine, Friedrich-Alexander-Universität Erlangen-Nürnberg).

## 3. Results

We analyzed eight PD patients, thereof two patients with adult onset, one patient with juvenile onset, two patients with childhood onset, and three patients with muscular infantile onset of PD. The patient collective is composed of three adult patients with milder disease forms (from 36 to 59 years) and five younger patients aged from 3 to 16 with a wide range of severity of symptoms (Table 1).

Two patients were able to walk independently; another two patients were supplied with walking aids (crutches). Powered wheelchairs were provided in 4 cases, whereof one patient was still able to use an active wheelchair for indoor activities. Three patients were dependent on ventilator support. Patient 4 and 5 were provided with intermittent noninvasive ventilation; patient 7 was ventilated via tracheostomy round the clock (Table 1).

### 3.1. Shoulder Girdle

Patient 3 presented with a winged scapula (scapula alata) as one of the first symptoms in childhood which revealed noticeable general weakness and led to the diagnosis of PD. The scapula alata remained regressive with continuous physiotherapy during a surveillance time of 10 years.

Patient 2 reported weakness in arms and shoulders at the age of about 40 years before developing weakness in the lower extremities. This patient presented with weakness and atrophy of the shoulder muscles, recurrent pain, and limited range of motion in the right shoulder at the age of 50 due to a rupture of the rotator cuff, tendinosis calcarea, and subacromial impingement. Surgery was carried out to reconstruct the right rotator cuff and for subacromial decompression. The patient had no physical complaint in the shoulder during one-year aftercare. ERT was planned to be started shortly after the last documented control examination.

Patient 7 reported recurrent subluxations of the left shoulder with occasional discomfort. Imaging and further examination were refused because of poor general condition of the patient.

### 3.2. Lower Extremities

Patient 1 suffered from progressive walking insecurity and pain in his hip and lumbar region at the age of around 45. Severe degenerative changes in the lumbar vertebral column, a hip joint with progressive dysplastic coxarthrosis, and subluxations were found. The constant pain, especially while walking, led to limited mobility and indicated total endoprosthetic (TEP) surgery of the left hip joint. The joint was secured with a cemented endoprosthesis with a snap cup in order to prevent further luxation. During postoperative care, the patient reported a decrease of pain. The remobilization was prolonged because the disease causes weakness of his hip stabilizing muscles; walking was possible with walking aids (crutches) after 21 days of hospitalization. Longer aftercare data is not available.

Patient 2 was diagnosed with PD after noticeable progressive leg weakness and difficulties with climbing stairs. The muscle strength remains sufficient with regular physiotherapy during the 14-month follow-up; further orthopedic treatment was not indicated.

Three of five patients with childhood PD form had severe hip dysplasia which led to hip joint surgery in two cases (Table 2). Patient 4 suffered from right hip dysplasia with recurrent subluxations because of the muscular imbalance. An intertrochanteric varisation osteotomy was performed and the patient was subsequently able to walk over short indoor distances. Further deterioration of the hip dysplasia or clinical restriction was not found during three-year aftercare. Patient 5 suffered from recurrent luxation on both sides and received a derotating varus osteotomy with hip and knee flexor release on both of the left and right sides. Walking was not possible before or after the surgery, but further luxation was avoided during the 10-year aftercare period. Patient 6 also showed hip dysplasia on both sides with a subluxated hip joint on the left side. Surgical treatment was not performed because of absence of clinical symptoms and severe insufficiency of the hip stabilizing muscles.

The patients with adult and juvenile forms in our collective were not affected by contractures of the lower extremities or foot deformities; however, it was a frequent finding in patients with childhood and muscular infantile PD forms. Two patients presented with distinct hip and knee joint contractures (patients 5 and 6) and four patients had feet deformities (patients 4, 5, 7, and 8) (Table 2).

Surgical correction of hip and knee contractures was performed in the two patients suffering from contractures of the hip and knee flexor muscles (patients 5 and 6). The surgical release of hip and knee flexor muscles was performed on both sides outside of our hospital with consecutive physiotherapy. Preoperative data was not available for patient 5, but the postoperatively achieved correction was found to be deteriorated when the patient was presented 10 years later to our consultation; contractures of 30° of both hip joints and 40° of both knee joints were measured at that time. Patient 6 had preoperative hip contractures of 50° left and 40° right and knee contractures of 25° on both sides which were corrected to 0° after surgery. During follow-up, no mobility limitations were found for the first four years, but contractures increased as of the fifth year to 15° left and 30° right of the hip and 40° left and 10° right of the knee. Night braces were not tolerated by the patient. Both patients were wheelchair bound.

Deformities of the feet were mostly flexible and treated conservatively with physiotherapy; additional surgical intervention was indicated only in one patient. Patient 8 underwent frontal achillotenotomy on both sides at the age of 5. Lower leg cast night splints were provided after surgery. Aftercare data is not available as the patient fails to appear to the check-up appointments.

### 3.3. Fractures

Patient 7 has been presented with a swollen thigh after sleeping; a spontaneous right supracondylar femur fracture, which was noticed because of autonomous bearing of body weight, independent sitting, standing, or walking, was never achieved. The fracture occurred without any known history of trauma and has been treated conservatively; clinical symptoms were not presented during 6-year aftercare.

### 3.4. Scoliosis

Six of our eight patients presented with scoliosis varying parameter values, depending on the severity of the disease. The patients with milder course of the disease (adult and juvenile forms) had a lower grade of scoliosis (less than 30°) which did not affect their mobility or respiratory function (patients 1 and 3). Both patients were provided with physiotherapy. Patient 3 had unaltered scoliosis at the 3-year follow-up. No follow-up data is available for patient 1.

Four of five patients with earlier PD onset suffered from severe scoliosis of 60°–90° and pelvic tilt ranged from 35° to 60° (Table 2). Patient 5 presented with a rigid spine phenomenon. Indication for surgical spine stabilization was given for these four patients because of progressive posture restriction and loss of sitting ability. Patient 4 was not able to undergo surgical intervention because of poor respiratory status and was therefore fitted with corset to stabilize his sitting position. Scoliosis severely deteriorated from 30° to 80° with pelvic tilt of 40° during four years. The patient was able to sit with the corset when holding on to something, for example, at the table; completely autonomous sitting was not achieved. It was not possible to balance his scoliosis when lying down. No surgical treatment was performed in patient 7 due to his extremely poor general condition, massive respiratory disorder, neurological problems, and very limited compliance of the family. Sitting ability was not achieved and the corset acceptance was very low.

Patients 5 and 6 were provided with surgical multisegmental dorsal spine stabilization. We used a Stryker XIA-System steel implant and similar operation technique in both cases. Pedicular screws were utilized from L1 to S1, transversal and laminar hook claw construction on Th3 and Th5, and double Luque wiring from Th6 to Th12. The vertebral joints between Th12 and S1 were opened on both sides. To support the stability after fixation, two additional cross connectors were used.

**Figure 1:**
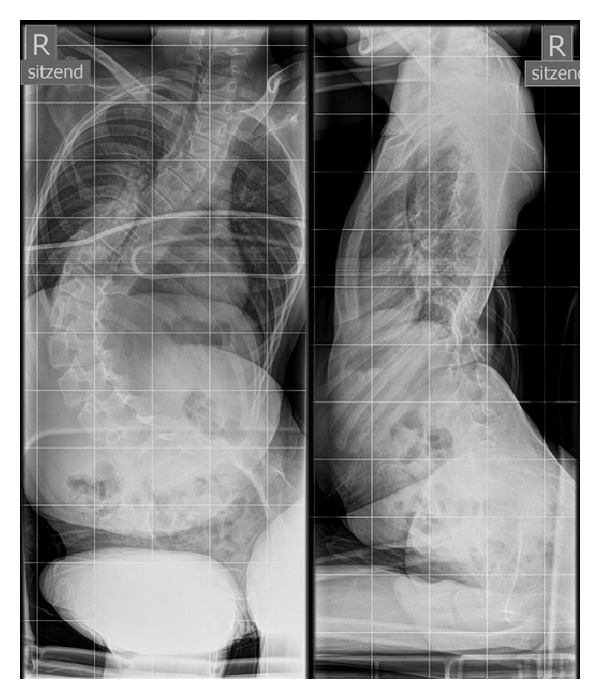
Male PD patient, 13 years old. Scoliosis right-convex C-shaped 90° Cobb Th6/L1/L5, before surgical spine stabilization. Pelvic obliquity 40° to the right. AP and sagittal radiograph.

**Figure 2:**
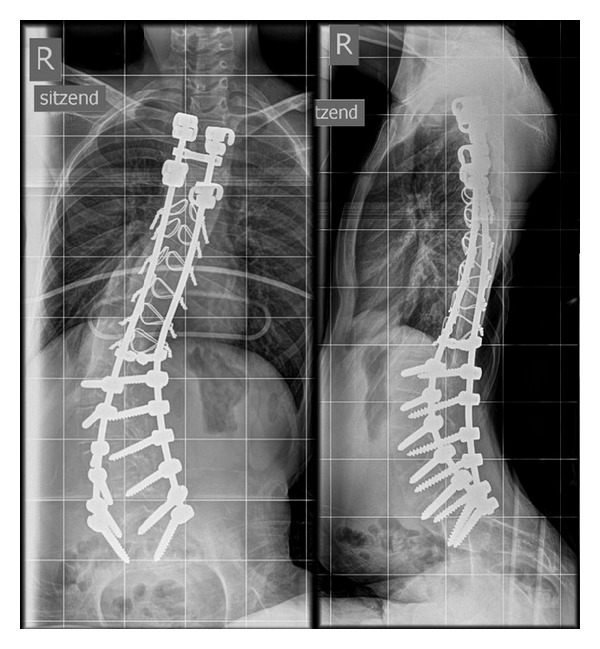
Male PD patient, 13 years old. Thoracic scoliosis 35° Cobb Th6/L1/L5, after surgical spine stabilization. Remaining 8° of pelvic obliquity to the right. AP and sagittal radiograph.

The postoperative period for patient 5 was inconspicuous. The thoracic right-convex part of scoliosis was corrected from 60° to 38° and the lumbar left-convex part from 60° to 23°. Pelvic tilt was improved from 35° to 0° (Table 2). Patient 6 suffered from disturbed wound healing which was discovered through crust formation and seroma during aftercare. The wound was revised a month later when the fascia was reopened and the Redon drainage was inserted. The outcome was not affected by the revision; the thoracolumbar right-convex scoliosis was corrected from 90° to 35° and the pelvic tilt was improved from 40° to 8° (Figures 1 and 2, Table 2). Both patients were able to sit independently after postoperative rehabilitation. Patient 5 underwent a follow-up examination after 7 months with stable scoliosis and pelvic tilt parameters; for further examination, the patient was not available. Patient 6 participated in regular control examinations; the two-year follow-up shows 37° scoliosis and 11° pelvic tilt which results in an annual loss of 1° of scoliosis and 1,5° of pelvic tilt. All measurements were performed on X-ray images in sitting position.

## 4. Discussion

As already mentioned above, PD is a very rare disease. Our patient collective is a selection of PD patients with orthopedic problems who applied to our neuromuscular disease consultations. The general muscle weakness in the PD course led to lower extremity disturbances including hip dislocations and subluxations, hip and knee flexor contractures, and feet deformities as well as shoulder-girdle involvement with less frequent and milder occurrence than lower limb disorders. Progressive scoliosis was found frequently with severe development during the disease course. These findings are characteristic for PD and in line with other publications [1, 13, 15].

Our small number of patients does not allow a general statement about the prevalence and therapy of orthopedic disorders in PD. Nevertheless, the present paper is the first to describe therapeutic approaches for the orthopedic treatment of scoliosis in PD patients.

Weakness in lower extremity and hip joint involvement is common in the progression of the disease [1] and usually manifested in delayed walking development in children and constricted standing and walking stability [13]. Two of the adult patients retrospectively noticed first muscle weakness in childhood. In adult PD form with slower progression of the disease, anamnesis often reveals early difficulties with climbing stairs, indicating an involvement of gluteal muscles and leading to progressive weakness of the hip joint muscles [1, 16]. This loss of muscular stability and muscular fixation is the reason for degenerative transformation of the hip joint and difficulty with climbing stairs is one of the first symptoms. As the disease takes its course, osteochondrosis and arthrosis lead to pain, so that posture worsens and the patient becomes less active. The lack of activity leads in turn to faster progression of muscle degeneration and aggravates the causal problem. Patients with sufficient muscle strength and walking ability seem to profit from surgical treatment, such as hip replacement.

In children, the muscle weakness in PD seems to have a strong negative effect on hip joint development [6]. Two of our patients had surgical hip joint correction to maintain walking or sitting stability, as already described elsewhere [13]. Surgical correction should be considered as a treatment option for hip disorders with restrictions on posture stability and mobility of PD patients.

Two of our patients firstly reported weakness in the upper extremities; a winged scapula led to the diagnosis of PD. The clinical manifestation of shoulder-girdle problems mostly appears later than the hip joint disorders, even though some cases with weakness of shoulder-girdle muscles as a first symptom of PD are described [1]. We assumed that the additional encroachment of the shoulder muscles in patient 2 was caused by the use of walking aids, since the muscles of the shoulder girdle were already weakened as a result of PD.

Four patients suffered from feet deformities which are frequently found in PD including pes equinus, talipes equinovarus, and pes planovalgus [13]. Another focus for orthopedic therapy is the frequently occurring contractures of hip- and knee-flexor muscles [1, 13] as presented in two patients in our case series. Adequate and timely physiotherapy is important and can eventually reduce the progression of the contractures [13, 17]. Contractures in our patients deteriorated in spite of physiotherapy; lower leg cast splints or night braces were not tolerated. Nevertheless, surgery remains an important option in case of manifested contractures or muscle shortening [13].

The spontaneous fracture of the femur as seen in one of our patients (patient 7) seems to be a known problem of PD. Similar cases of spontaneous fractures without known trauma history or independent sitting or walking have been described [18]. Fractures of the femur were investigated as the most common fracture in children with PD [18].

Some publications in recent years have explored the effect of low bone mass density probably caused by PD [1, 14, 18–20]. It was shown that already at a young age patients commonly developed a very low bone density with a high risk of long bone fracture, especially of the femur bone. There seems to be a correlation between low bone density and decreased proximal muscle strength and it is a particular risk for wheelchair-bound and ventilator-dependent patients, such as our patients [14, 19].

Severe scoliosis was the most frequent and most prominent finding within our patients. It has already been shown elsewhere that scoliosis is more frequent in wheelchair-bound patients and the progress of the disease is faster after loss of ambulation [15]. It has furthermore been shown that scoliosis occurs more frequently in individuals with the childhood form of PD [13, 15] and is often associated with impaired respiratory function [15]. Scoliosis is a significant problem in PD and needs to be examined closely in order to initiate a therapy in time, especially after increased survival rates due to ERT [13, 15].

Two patients underwent surgical spine stabilization and showed positive effects after the intervention concerning the radiographical measure of scoliosis as well as posture and sitting stability. However, the diversity of the forms of PD is very large which makes it difficult to draw a general conclusion. Further investigation with a larger number of patients is necessary to evidence the positive effect of spinal surgery on scoliosis and the general status of PD patients. We can conclude from our experience that spinal surgery can be an option for scoliosis treatment in infantile and late-onset PD patients and should be considered as a therapy of choice in higher degree scoliosis. Furthermore, we observed no specific surgical or anesthetic complications during operations or anesthesia [21]. The rigid spine phenomenon, as presented in one of our patients, should be kept in mind as a possible symptom in the phenotypic presentation of PD. Two other cases of rigid spine in PD were recently described in the literature with the conclusion that the rigidity of the spine accompanies contractures of limb joints [22–24].

The course of PD is changed by ERT, leading to skeletal muscle pathology with additional symptoms [25] and impact on the musculoskeletal system, making orthopedics more relevant [2, 13, 26]. Although surgical interventions are gaining importance especially for young PD patients, physiotherapy remains a very important part of the multidisciplinary therapeutic approach [6, 13, 14]. Regular physiotherapeutic treatment should stabilize skeletal muscle, improve muscle function, and prevent secondary musculoskeletal impairments [14, 17]. Respiratory training can improve respiratory muscle function and pulmonary status [14, 27] which should regularly be examined in order to provide early ventilator support [28] like the three patients in the present paper. Additional exercises are recommended, as a certain amount of muscle activity has doubtlessly a positive effect on the course of the disease [6, 13]. Orthopedic technical devices are frequently used in management of PD patients in order to provide passive stretching and posture benefits, to support the independent activity, and to stabilize the sitting position [1, 13, 14].

## 5. Limitations

The present paper has some limitations due to small number of patients, the retrospective data collection, and very irregular and nonstandardized follow-up data available. It remains a descriptive case series for a limited number of orthopedic problems. Since publications for the orthopedic surgical treatment for PD patients are not available at present, further analysis of the therapeutic options is required.

## 6. Conclusion

Orthopedic management gains importance due to extended survival and musculoskeletal involvement under ERT. Continuous care and follow-up is important in order to identify musculoskeletal problems at an early stage and provide a timely and adequate treatment. In order to improve the quality of life, in some cases, surgical treatment as enhancement of the conservative therapy can be indicated. The present paper offers the first description of surgically treated scoliosis and therapy options for continuative orthopedic problems in PD patients. Further evidence for the surgical spine stabilization for PD patients remains to be shown with larger number of patients.

## Figures and Tables

**Table 1:** Patient details.

Pat.	Sex	Age^1^ (years)	Height/ weight^1^ (cm/kg)	Follow-up^2^	PD type	Age of PD diagnosis (years)	Age of ERT beginning (years)	Respiratory involvement/vital capacity	Ventilatory support (time/day)	Movement support
1	F	59	170/58	—	Adult LOPD	51	No	No	—	Crutches
2	M	50	167/57	14 months	Adult LOPD	44	Planned	Yes/IVC 48%	—	Crutches
3	M	36	186/60	11 years	Juvenile LOPD	13	32	Yes/IVC 40%	—	—
4	M	16	176/30	3 years	Childhood LOPD	2	No	Yes/IVC 37%	NIV (16 h)	AWC, PWC
5	M	12	140/18	19 months	Muscular IOPD	1^3^	6	Yes/IVC 30%	NIV (night)	PWC
6	M	3	155/33	12 years	Muscular IOPD	1^3^	10	Yes/IVC 32%	—	AWC, PWC
7	M	5	144/30	6 years	Muscular IOPD	1^3^	3	Yes	TrS (24 h)	PWC
8	M	4	166/15	—	Childhood LOPD	4	4	No	—	—

Pat.: patient, F: female, M: male, NIV: noninvasive ventilation, TrS: tracheostoma, PWC: powered wheelchair, and AWC: active wheelchair.

^1^Age, height, and weight at first presentation in our neuromuscular disease center. ^2^Time period of continuous control investigations during our consultations. ^3^Within first year of life.

**Table 2:** Orthopedic findings and treatment.

Pat.	First symptoms	Sitting ability	Walking ability	Scoliosis (Cobb degree)	Pelvic tilt	Hip joint involvement contractures, feet deformities	Orthopedic surgeries	Age of surgery (years)
1	LW	Yes	Yes	Incipient, <10°	—	—	Hip-TEP left	59
2	LW	Yes	Yes	—	—	—	Reconstruction of rotator cuff right	50
3	GW	Yes	Yes	Th: right-conv. 26° Th3/Th7/Th11	—	—	—	—
4	IMD	No	Limited^2^	Th/L: right-conv. C-shaped 80° Th5/Th11/L5	40° right	Hip dysplasia/luxation right Pes equinus	Intertrochanteric varus osteotomy right	17
5	IMD	Yes^1^	No	Th: right-conv. 57° Th1/Th8/Th12^4^; L: left-conv. 90° L1/L3/L5^4^	35° left	Hip dysplasia/luxation left + right Hip + knee flexor contractures Pes planovalgus	Spine stabilization Th3-S1 Post-op: Th 38°, L 23°, pelvic tilt 0°	14
							Derotating varus osteotomy both sides; hip and knee flexor release left+right	13
6	LW	Yes	No^3^	Th/L: right-conv. C-shaped 90° Th6/L1/L5	40° right	Hip dysplasia left + right Hip + knee flexor contractures	Spine stabilization Th3-S1 Post-op: Th 35°, pelvic tilt 8°	13
							Hip and knees flexor release left + right	4
7	LW	No	No	Th/L: left-conv., collapsing spine^5^	60° left	Talipes equinovarus	Surgery not possible^6^	—
8	IMD	Yes	Yes	—	—	Pes equinus	Achillotenotomy left + right	5

Pat.: patient, LW: leg weakness, GW: general weakness, IMD: impaired motor development, left-conv.: left-convex, right-conv.: right-convex, Th: thoracic, and L: lumbar. ^1^was lost due to scoliosis and pelvic obliquity and regained after spine stabilization surgery. ^2^Only transfer walking for a few meters inside. ^3^Ability lost at the age of 13 years. ^4^Last examination before surgery. ^5^Cobb degree not measurable and sitting position for X-ray is not achievable. ^6^Operative stabilization was indicated; patient's general condition is not sufficient for surgery.
